# Functional Printing of Conductive Silver-Nanowire Photopolymer Composites

**DOI:** 10.1038/s41598-019-42841-3

**Published:** 2019-04-23

**Authors:** Tomke E. Glier, Lewis Akinsinde, Malwin Paufler, Ferdinand Otto, Maryam Hashemi, Lukas Grote, Lukas Daams, Gerd Neuber, Benjamin Grimm-Lebsanft, Florian Biebl, Dieter Rukser, Milena Lippmann, Wiebke Ohm, Matthias Schwartzkopf, Calvin J. Brett, Toru Matsuyama, Stephan V. Roth, Michael Rübhausen

**Affiliations:** 1 Institut für Nanostruktur- und Festkörperphysik, Center for Free Electron Laser Science (CFEL), Universität Hamburg, Luruper Chaussee 149, 22761 Hamburg, Germany; 2 DESY, Notkestrasse 85, 22607 Hamburg, Germany; 3 Department of Mechanics, KTH Royal Institute of Technology, Teknikringen 8, 100 44 Stockholm, Sweden; 4 Wallenberg Wood Science Center, Teknikringen 56-58, 100 44 Stockholm, Sweden; 5 Max-Planck Institute for the Structure and Dynamics of Matter, Luruper Chaussee 149, 22761 Hamburg, Germany; 6 Department of Fiber and Polymertechnology, KTH Royal Institute of Technology, Teknikringen 56-58, 100 44 Stockholm, Sweden

**Keywords:** Nanowires, Nanocomposites

## Abstract

We investigated the fabrication and functional behaviour of conductive silver-nanowire-polymer composites for prospective use in printing applications. Silver-nanowires with an aspect ratio of up to 1000 were synthesized using the polyol route and embedded in a UV-curable and printable polymer matrix. Sheet resistances in the composites down to 13 Ω/sq at an optical transmission of about 90% were accomplished. The silver-nanowire composite morphology and network structure was investigated by electron microscopy, atomic force microscopy, profilometry, ellipsometry as well as surface sensitive X-ray scattering. By implementing different printing applications, we demonstrate that our silver nanowires can be used in different polymer composites. On the one hand, we used a tough composite for a 2D-printed film as top contact on a solar cell. On the other hand, a flexible composite was applied for a 3D-printed flexible capacitor.

## Introduction

Functional printing of films and additive manufacturing of components have developed over the last 25 years to be an important and innovative part of the industrial process^1^. From a scientific point of view, the limited physical properties of polymers used mainly for 3D printing represent one of the largest challenges^2^. For electronic applications, conductive layers with good printability are of key importance. Conductive polymers such as PEDOT:PSS have been used for conductive layers^3–5^. Functionalized composite resins are an important alternative as they are also photopolymers and therefore 3D-printable. In this case, the polymer matrix determines the structural and mechanical properties that can be tuned e.g. from tough to flexible. Composites of spherical nanoparticles have to overcome the tunnelling resistance when electrons move from one metallic nanoparticle to the next. Hence, previous approaches to establish resins with good conductivity based on spherical nanoparticles suffer from problems of agglomeration and strong photon absorption at high nanoparticle concentrations. Therefore, they are often unsuitable for optical applications requiring transparent contacts^6,7^.

Nanowires offer an alternative approach, which circumvents the tunnelling resistance in the direction of the wire. Silver-nanowire (Ag-NW) electrodes can be realized by various routes, including printing and spray-coating^8–11^. Ag-NW composites offer a scalable process for large scale, flexible, conductive media, as used in e.g. integrated photovoltaics, touch screens, and flexible electronics^12–19^. The plasmonic effects of metallic nanoparticles and nanowires render them useful as advanced optoelectronic devices and biosensors^20–25^. The sheet resistances of spray-coated Ag-NW layers are between 10 Ω/sq and 30 Ω/sq at transparencies of around 90%^10,26^. Thus, they are potential candidates for replacing indium tin oxide in, for example, solar cells^27^. Due to their high aspect ratio, ordered arrangements are possible, leading to an anisotropic conductive behavior^28,29^. Furthermore, the development of flexible electronics based on silver nanowires is progressing^30–34^. Printable flexible Ag-NW composites enable the fabrication of flexible electronic devices with complex geometric shapes, rapid prototyping, and high design flexibility due to additive manufacturing^1,35^. Therefore, we study photopolymer-based silver-nanowire composites. For characterization, we utilized grazing incidence small angle X-ray scattering (GISAXS), scanning electron microscopy (SEM), profilometry, optical transmission, electrical conductivity measurements, energy dispersive X-ray spectroscopy (EDX), atomic force microscopy (AFM), and ellipsometry. By using a tough and a flexible polymer matrix, we demonstrate the application of our Ag-NW composites as a solar cell top contact and as a 3D-printed flexible capacitor.

## Results and Discussion

**Figure 1:**
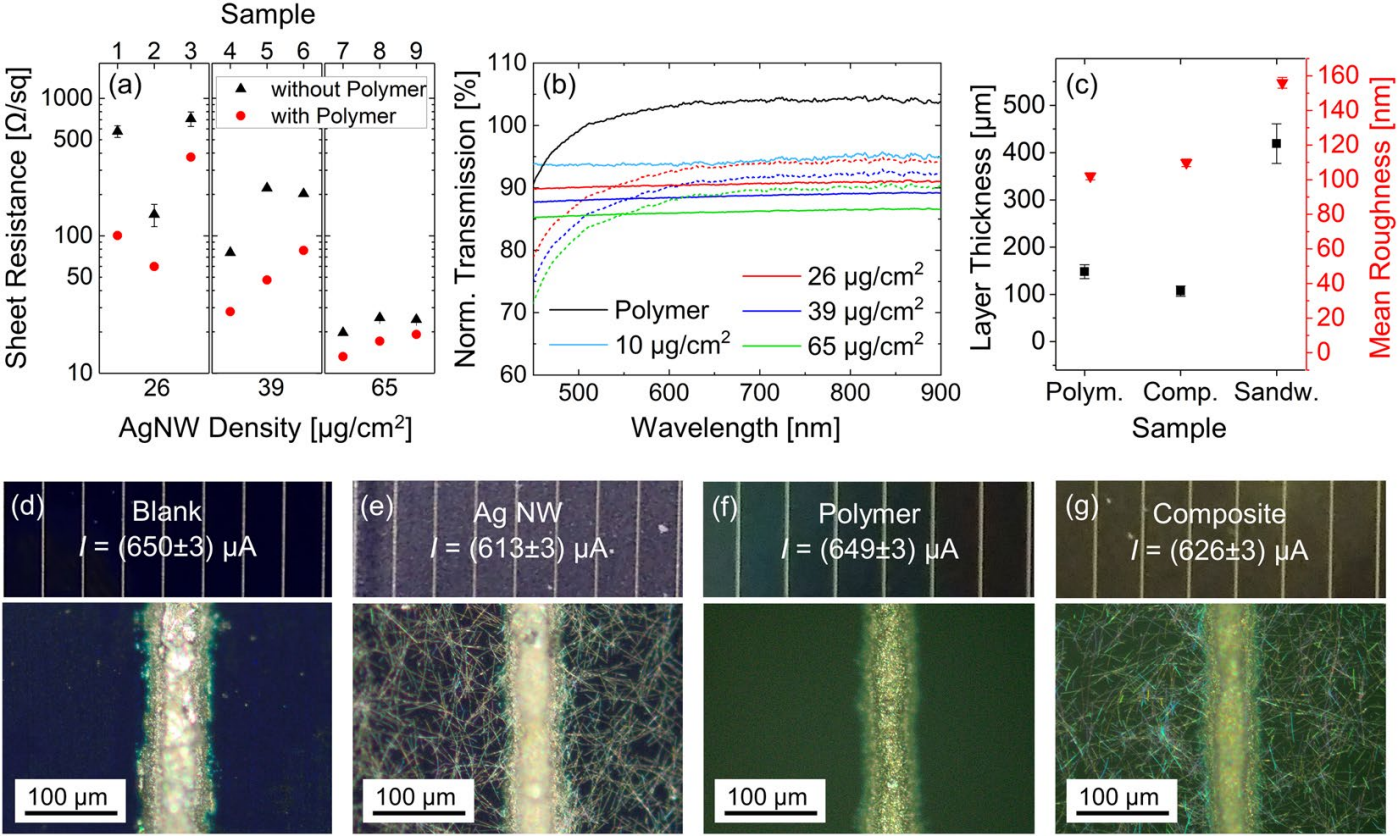
Characterization and 2D printing of the tough Ag-NW-polymer composite. (**a**) Impact of the polymer crosslinking on the Ag-NW composite conductivity by comparing the sheet resistances of Ag-NW networks (black dots) and Ag-NW composites (red dots) for 3 different Ag-NW densities (26 µg/cm^2^, 39 µg/cm^2^ and 65 µg/cm^2^). At high Ag-NW densities, the reproducibility of the measured sheet resistances is enhanced, but the impact of the polymer coating on the conductivity is reduced. (**b**) Transmission of visible to near infrared light through Ag-NWs and Ag-NW composites. Solid lines indicate pure polymer or Ag-NW films, whereas dashed lines indicate Ag-NW composites. The transmission is larger than 87% for all composites between 600 nm and 800 nm normalized to a bare glass substrate. The polymer coating decreases scattering and reflection at the glass interface resulting in an enhanced transmission compared to a bare glass slide. (**c**) Exemplary layer thickness and roughness of the produced polymer samples: pure polymer layer, Ag-NW (7 µg/cm^2^) composite and polymer-Ag-NW (22 µg/cm^2^)-polymer multilayer sample. Please note that layer thicknesses between 20–300 µm represent typical thicknesses in functional printing. The thickness to surface roughness ratio is >1000:1. (**d**) Photograph and optical microscopy image of a blank solar cell (monocrystalline, 60010, Sol-Expert). A photocurrent *I* of 650 µA was measured during exposure with an Ulbricht sphere. (**e**–**g**) Photographs, optical microscopy images and measured photocurrents of coated solar cells ((**e**) Ag-NWs, (**f**) polymer, (**g**) composite).

We demonstrate the application of our Ag-NWs within a tough polymer matrix as transparent and conductive top contact in Fig. 1. Figure 1(a) shows the conductivities of the Ag-NW networks and the corresponding composites after photopolymerization. The samples were prepared by coating of an Ag-NW layer with 1,6-Hexanediol diacrylate (HDDA) based resin (see Supplementary Information S1.4) and curing with UV-light (see Supplementary Information S1.5). The HDDA-based polymer matrix has a resistance of above 1 MΩ. We synthesized the Ag-NWs *via* the polyol route^36,37^ resulting in nanowires with high aspect ratios (length/diameter) between 100 and up to 1000. The influence of the polymer layer on the conductivity of the nanowire network is shown in Fig. 1(a). The Ag-NW network alone has a decreasing sheet resistance with increasing nanowire concentration from about 500 Ω/sq at 26 µg/cm^2^ down to 13 Ω/sq at 65 µg/cm^2^. The polymer deposition and cross-linking have a positive impact on the conductivity. It is known that resins compress during the curing procedure^38,39^. Shrinking of the polymer matrix presumably also compresses the nanowire network upon polymerization. This decreases the sheet resistance close to the critical nanowire concentration by a factor of about 2–5 as can be seen in Fig. 1(a). The impact of the compression decreases with increasing nanowire concentration from several 100% at 26 µg/cm^2^ to a few percent at 65 µg/cm^2^. The reason is the already enhanced nanowire density resulting in higher conductivities at higher concentrations. Reducing the nanowire density leads also to a higher sensitivity on how the nanowires connect to form a conductive network (see Supplementary Fig. S9).

The transmission spectra of bare Ag-NW networks and Ag-NW composites in reference to a bare glass substrate are shown in Fig. 1(b). At a nanowire concentration of 26 µg/cm^2^ of the composite, we find a transmission of 94% at 700 nm and a sheet resistance of up to several 100 Ω/sq. At 65 µg/cm^2^ the transmission drops to about 90% and the sheet resistance decreases down to 13 Ω/sq. These results are compatible with previous studies^40–43^. Due to its intermediate refractive index, the bare polymer already enhances the transmission through the glass substrate. As expected, we observe a reduced transmittance with increasing nanowire concentration. The relevance of optimizing the conductivities at relatively low nanowire concentrations, where the compressive effects of the composite are the largest, becomes apparent. This indicates the delicate interplay between the optimization of the polymer matrix and the Ag-NW network when an application is intended as a transparent top contact.

The typical layer thicknesses used in our study are in agreement with typical layer thicknesses used for functional printing ranging between 20 and 300 µm. For optical applications, a control of the surface roughness is of high relevance. Figure 1(c) depicts the layer thickness and surface roughness as determined by profilometry of the polymer, the Ag-NW composite, and a multilayer structure. The latter serves as model for 3D-printed multilayers, which places an Ag-NW layer between different polymer layers. Remarkably, the surface roughness is about 110–160 nm for the Ag-NW composite materials, which is primarily determined by the roughness of the polymer. The thickness to surface roughness ratio is about 1000. Thus, we can expect reduced losses due to diffuse scattering effects. These results show that the composite Ag-NW-polymer materials can act as competitive materials for conducting and light-transparent electrodes.

In order to demonstrate an application of the composite as potential top contact we have printed a conductive Ag-NW composite film (26 µg/cm^2^) on top of a monocrystalline solar cell. For comparative purposes, we have also printed a bare polymer layer and a bare conductive Ag-NW-network. Figure 1(d–g) shows the blank solar cell (d), the Ag-NW coated solar cell (e), the polymer coated solar cell (f), and the solar cell coated with the Ag-NW composite (g) with their corresponding photocurrents under identical illumination conditions. The photocurrent decreases only slightly from the blank solar cell ((650 ± 3) µA) to the Ag-NW composite ((626 ± 3) µA). The composite showed a better transmission than the pure Ag-NW layer due to better phase index matching according to Fig. 1(b). Overall, the use of an Ag-NW-polymer electrode on top of the solar cell resulted in a 4% decrease of the measured current. The sheet resistance in the particular configuration of the Ag-NW network was (75.2 ± 1.7) Ω/sq and of the Ag-NW composite was (63.0 ± 3.5) Ω/sq, also in agreement with Fig. 1(a).

**Figure 2:**
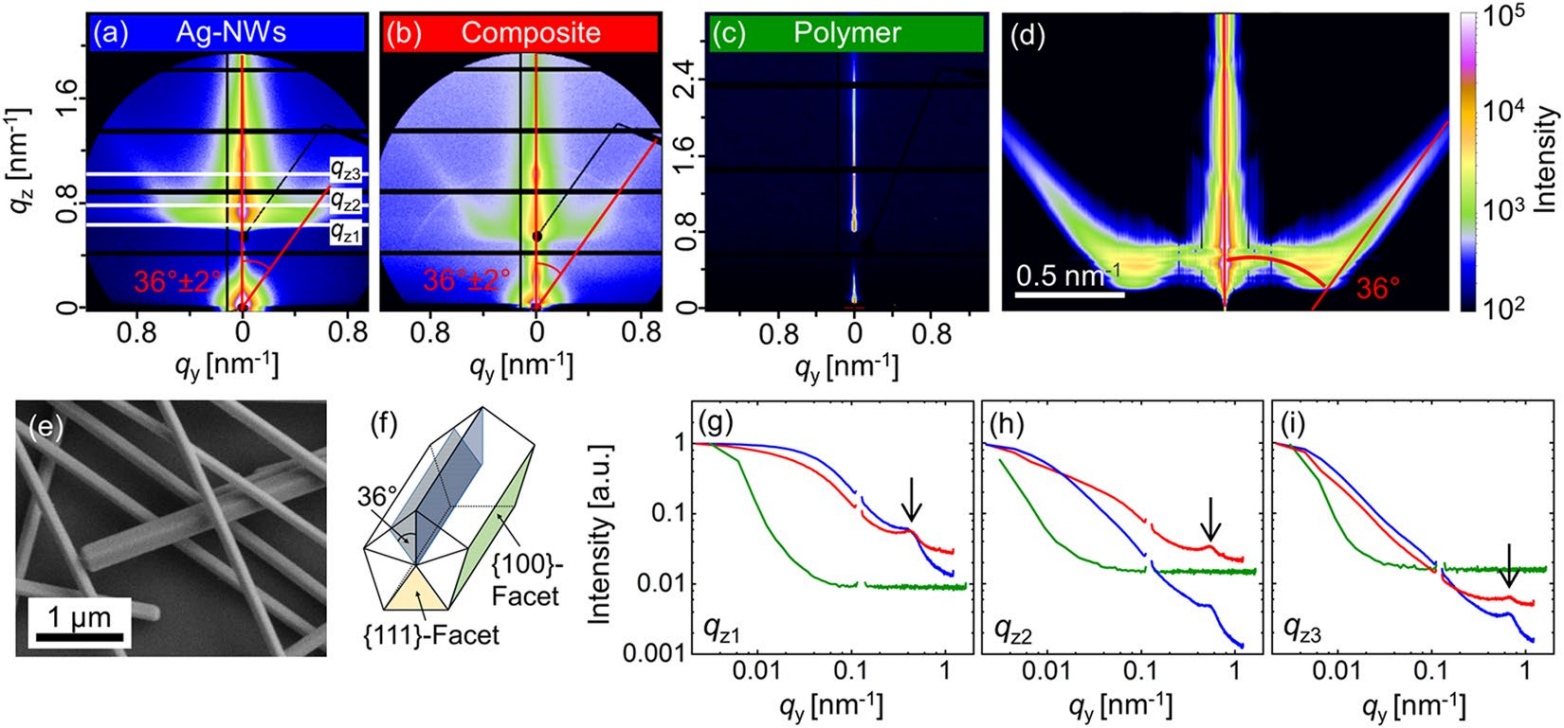
Two-dimensional (2D) GISAXS pattern from the samples. (**a**) Ag-NWs (58 µg/cm^2^). (**b**) Ag-NWs (7 µg/cm^2^) coated with UV-cured polymer layer. (**c**) Bare UV-cured polymer. Intensity scale bar is shown on the right side. Clear flares at 36° ± 2° (indicated by red lines) stemming from the facets of the pentagon morphology starting from the sample horizon are visible for (**a**,**b**). (**d**) Simulation of the key scattering features of Ag-NWs. (**e**) SEM image of Ag-NWs on a silicon substrate (Ag-NW density around 120 µg/cm^2^). (**f**) Sketch of the faceted Ag-NW (adapted from^44,45^). (**g**) Horizontal cuts of the intensity (*I*(*q*_y_,*q*_z1_ = 0.63 nm^−1^)), (**h**) *I*(*q*_y_,*q*_z2_ = 0.78 nm^−1^), (**i**) *I*(*q*_y_,*q*_z3_ = 0.96 nm^−1^). All cuts are normalized to the intensity at *I*(0,*q*_z1,2,3_) correspondingly. The colour code corresponds to (**a**–**c**).

In order to characterize the Ag-NW composites we apply grazing incidence small angle X-ray scattering (GISAXS). GISAXS enables the investigation of structural properties of the Ag-NW network embedded in the HDDA-based polymer matrix with statistical relevance. The results are summarized in Fig. 2 and the following samples were investigated: Ag-NWs (Fig. 2(a)), the Ag-NW composite (Fig. 2(b)), and the pure polymer layer (Fig. 2(c)). As reference substrate, a silicon wafer was used. The polymer pattern of Fig. 2(c) is essentially featureless. In Fig. 2(a) and (b) two intensity flares with an angle of around 36° to the vertical direction can be seen outlining a contribution from the Ag-NWs. Figure 2(g–i) depict horizontal cuts at different *q*_z_ positions for the different samples (green polymer, blue Ag-NW network, and red Ag-NW composite, compare Fig. 2(a–c)) as indicated by the white lines in Fig. 2(a). A side maximum in the region 0.4–0.8 nm^−1^ occurs when a flare is present in the GISAXS pattern (labelled with an arrow in Fig. 2(g–i)). The shift corresponds to the facet angle of around 36°. The polymer coating decreases the scattering contrast between the Ag-NWs and the surrounding medium, thus at higher *q*_z_ the peak of the Ag-NW polymer composite appears smaller.

To understand the origin of the flares, Fig. 2(f) depicts a sketch of the Ag-NW with pentagonal cross-section^44,45^. Ag-NWs, synthesized *via* the polyol method, exhibit a pentagonal structure. In the early state of the synthesis, five-fold twinned seeds are formed, which grow in <110> direction^46^. The pentagonal structure and fivefold twinned tip of our Ag-NWs were also verified by SEM studies (Fig. 2(e)). However, SEM studies are not applicable to the composite. The pentagonal morphology is confirmed by simulating the key features of the observed GISAXS pattern using the software IsGISAXS^47^ (for details of the modelling, see Supplementary Information S2.6), as shown in Fig. 2(d). The deduced angle of the intensity flares is 36° and compares well with the facets of the pentagonal morphology. Additionally, the enhancement of the diffuse scattering around the Yoneda peak (*α*_c_ = 0.14° for Si) can also be reproduced.

**Figure 3:**
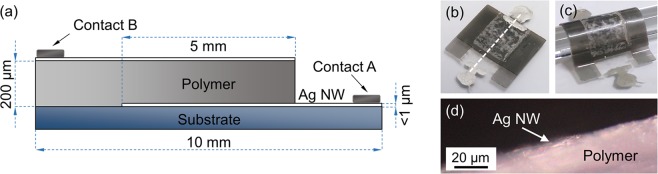
Flexible Ag-NW composite capacitor. (**a**) Illustration of the cross-section of the capacitor. (**b**) Photograph of a produced Ag-NW capacitor (10 × 10 mm^2^). The dashed white line depicts the position of the cross-section, which is presented in (**a**). (**c**) Photograph of the capacitor bent over a glass rod in order to demonstrate its flexibility. (**d**) Cross-sectional view of the lower part of the stripped off capacitor with Ag-NWs.

The further development of the 2D functional printing into the third dimension is fairly straightforward and can be accomplished within a 3D printing process. A capacitor consisting of Ag-NWs and a flexible photopolymer (Flexible, Formlabs) was prepared in order to demonstrate the feasibility of our approach for 3D-printed flexible electronics. It also highlights the relevance of the polymer of composite for improving the functional properties of the printed object. In order to print the capacitor, six layers along the third dimension were used. A glass slide was coated with resin. The resin and the composite were illuminated and cured by using a combination of three proximity illumination patterns with an area of 10 × 10 mm^2^. The remaining uncured resin was washed off with acetone and isopropanol. An illustration of the cross-section and a photograph of the resulting capacitor with two conductive Ag-NW composite plates and a resin dielectric are shown in Fig. 3(a,b). In order to contact the capacitor two silver-lacquer contacts were applied. The produced capacitor is made up of two 5 × 5 mm^2^ Ag-NW layers with an Ag-NW density of about 100 µg/cm^2^. In Fig. 3(c) a photograph of our stripped off capacitor bent over a glass rod is shown in order to demonstrate its flexibility. A total layer thickness of the intermediate polymer layer of (210 ± 5) µm was found by optical microscopy. Figure 3(d) shows an optical microscopic cross-sectional view of the capacitor with Ag-NWs at the surface.

The capacitance was estimated according to Eq. (1), where *ε*_0_ is the vacuum permittivity, *ε*_*r*_ is the polymer permittivity, *A* is the area of the capacitor and *d* is the layer thickness of the intermediate polymer layer. The estimated capacitance is 5 pF taking the dielectric constant to about 4.5. In good agreement with the estimation, a capacitance of 7.0 and 7.5 pF was reproducibly measured at 1 kHz and at 100 kHz, respectively.

1 $$C={\varepsilon }_{0}{\varepsilon }_{r}\times \frac{A}{d}$$

## Experimental Section

### Synthesis

We synthesized the Ag-NWs *via* the polyol route^36,37^. The used chemicals are listed in Table 1. Initially, an oil bath was heated up to 165 °C or 155 °C. In an unsealed vial, 6 mL of ethylene glycol (EG) was heated for 50 min in the oil bath and stirred at 600 rpm. In the meantime, 0.03 g silver nitrate (AgNO_3_) was added to 2 mL EG and polyvinylpyrrolidone (PVP) (0.03 g PVP 55000 + 0.06 g PVP 360000) was dissolved in 3 mL EG. After complete dissolution of the PVP, 3 µL copper(II) chloride (CuCl_2_) solution (*c* = 0.0519 mol/L containing CuCl_2_ ∙ 2H_2_O + 2 mL EG) was added to the PVP solution. After 50 min preheating, PVP in solution was added to the preheated EG and was heated further for 15 min. The solution was stirred at 600 rpm. The AgNO_3_ solution was injected into the reaction solution using a syringe pump (neMESYS, CETONI GmbH) and a flow rate of 1.88 mL/h. No stirring was applied during the addition of AgNO_3_. The reaction was quenched with 16 °C water after 180 min (165 °C) or 235 min (155 °C). After synthesis, the Ag-NWs were washed consecutively three times with acetone and three times with isopropanol. The suspension was centrifuged at 200 rpm at 20 °C for 20 min each time.

**Table 1:** Chemicals used for silver-nanowire synthesis.

Chemical			Amount
Ethylene glycol	EG	anhydrous, 99.8% purity, S. Aldrich	*V*_total_ = 11 mL, *n* = 0.20 mol
Silver nitrate	AgNO_3_	>99.9% purity, Carl Roth	*m* = 0.03 g, *n* = 0.18 mmol
Polyvinylpyrrolidone	PVP 55000 MW	Sigma Aldrich	*m* = 0.03 g, *n* = 0.55 µmol
	PVP 360000 MW	Carl Roth	*m* = 0.06 g, *n* = 0.17 µmol
Copper(II) chloride	CuCl_2_	99.999% purity, Sigma Aldrich	*V* = 3 µL of solution (*c* = 0.0519 mol/L), *n* = 0.156 µmol

### Sample preparation

For the sample preparation, cleaned substrates either of silicon or glass (see Supplementary Information S1.1) were used. Pure Ag-NW networks were fabricated by deposition of a drop of nanowire suspension in isopropyl alcohol leading to randomly orientated Ag-NWs forming a percolation network with a nanowire surface density between 6.5–120 µg/cm^2^ (see Supplementary Information S1.2). To fabricate the polymer films, 10 µL of a 1,6-Hexanediol diacrylate (HDDA) based resin (see Supplementary Information S1.4) or a flexible resin (Formlabs) was dropped on the surface of a cleaned substrate. The resin was cross-linked by illumination with a laser diode (*λ* = 405 nm, see Supplementary Information S1.5) for 10 min. The fabrication process of a composite sample combines the Ag-NW network and a polymer layer. Firstly, an Ag-NW network was produced. Secondly, the Ag-NWs were coated with resin, which was finally cured with UV-light. Multilayer structures were fabricated by a three-step process with an Ag-NW layer between two polymer layers. A summary of the composite materials and their key properties is given in the Supplementary Table S2.

### Characterization methods

For scanning electron microscopy measurements, a commercial field emission scanning electron microscopy (FE-SEM) equipment (Zeiss, Germany) was used (see Supplementary Information S2.1). Resistivity was determined in Van der Pauw^48^ geometry by utilizing a four probe measurement setup with DPP 105-M/V-Al-S positioners (CascadeMicrotech, USA) (see Supplementary Information S2.3). Transmission spectra were recorded with an Ulbricht sphere of calibrated spectral characteristics (DKD, Gigahertz Optik) coupled to a QE65000 spectrometer (Ocean optics, USA) in a spectral range between 400 nm and 900 nm. In order to measure the photocurrent of a coated solar cell, the same light source was used. A circular area with a diameter of approximately 5 mm was exposed with a power of about 20 mW/cm^2^, and the setup was sealed off from ambient light. The photocurrent was measured with a digital multimeter (Voltcraft, VC 850). Profilometry measurements were performed with a Dektak XT equipment (Bruker, USA). The capacitance of our flexible capacitor was experimentally determined with a programmable HM8118 LCR bridge (Rohde & Schwarz, Germany). GISAXS^49–51^ measurements were performed at the beamline P03 at PETRA III @ DESY^52^ with a wavelength of 0.972 Å. The sample-to-detector distance (SDD) was SDD = (4990 ± 1) mm calibrated using silver behenate, and the beam size (horizontal × vertical) was 25 × 15 µm^2^. The polymer sample was measured at an SDD of (3600 ± 1) mm with a beam size of 30 × 36  µm^2^. For detection, a 2D Pilatus 1 M (Dectris Ltd.) detector was used (981 × 1043 pixels, pixel size 172 µm). The flight path between sample and detector was evacuated to reduce scattering. An incident angle of around 0.5° was chosen (see Supplementary Information S2.5). Data reduction was performed using the software package DPDAK^53^. Our methods were selected such that the nanowire network in functional polymer-Ag-NW composites can be studied towards its critical parameters, namely network interconnectivity, optical transmission, sheet resistance, as well as thickness, and roughness.

## Conclusion

We have manufactured conductive silver-nanowire (Ag-NW) networks with use in functional printing. By applying two different polymers, we have fabricated composites with different properties that were tested for two specific applications. Firstly, we have optimized Ag-NW composites for use as transparent top contacts by tuning the Ag-NW concentration within a tough and transparent HDDA-based polymer matrix. We have accomplished a sheet resistance of 13 Ω/sq and a corresponding transmission at 700 nm of 90%. Secondly, we have used a flexible polymer matrix in the composite for a 3D-printed flexible capacitor. The capacity of around 7 pF agrees well with the estimated value of about 5 pF. Our characterization involves GISAXS, which enables the investigation of embedded nanostructures and interfaces with high statistical relevance. This shows that GISAXS can develop further to an excellent technique for the investigation of embedded nanostructures in 3D-printed and technically relevant films.

## Supplementary information

Supplementary Information

## Data Availability

The data of this study are available within the article and its Supplementary Information.

## References

[1] MacDonald E, Wicker R (2016). Multiprocess 3D printing for increasing component functionality. Science.

[2] Hofmann M (2014). 3D printing gets a boost and opportunities with polymer materials. ACS Macro Lett..

[3] Crispin X (2006). The Origin of the High Conductivity of Poly(3,4-ethylenedioxythiophene)−Poly(styrenesulfonate) (PEDOT−PSS) Plastic Electrodes. Chem. Mater..

[4] Frackowiak E, Khomenko V, Jurewicz K, Lota K, Béguin F (2006). Supercapacitors based on conducting polymers/nanotubes composites. in. Journal of Power Sources.

[5] Huynh WU, Dittmer JJ, Alivisatos AP (2002). Hybrid nanorod-polymer solar cells. Science (80-.)..

[6] Gonon P, Boudefel A (2006). Electrical properties of epoxy/silver nanocomposites. J. Appl. Phys..

[7] Nam S (2011). Effects of silica particles on the electrical percolation threshold and thermomechanical properties of epoxy/silver nanocomposites. Appl. Phys. Lett..

[8] Park JS, Kim T, Kim WS (2017). Conductive Cellulose Composites with Low Percolation Threshold for 3D Printed Electronics. Sci. Rep..

[9] Choi HW, Zhou T, Singh M, Jabbour GE (2015). Recent developments and directions in printed nanomaterials. Nanoscale.

[10] Ding Z, Stoichkov V, Horie M, Brousseau E, Kettle J (2016). Spray coated silver nanowires as transparent electrodes in OPVs for Building Integrated Photovoltaics applications. Sol. Energy Mater. Sol. Cells.

[11] Cui Z, Han Y, Huang Q, Dong J, Zhu Y (2018). Electrohydrodynamic printing of silver nanowires for flexible and stretchable electronics. Nanoscale.

[12] Hemmati S, Barkey DP, Gupta N, Banfield R (2015). Synthesis and Characterization of Silver Nanowire Suspensions for Printable Conductive Media. ECS J. Solid State Sci. Technol..

[13] He X (2017). Transparent electrode based on silver nanowires and polyimide for film heater and flexible solar cell. Materials (Basel)..

[14] Park JS, Kim BJ, Park JS, Hwang YJ (2015). Characteristics of silver meshes coated with carbon nanotubes via spray-coating and electrophoretic deposition for touch screen panels. in. Thin Solid Films.

[15] Leem D-S (2011). Efficient Organic Solar Cells with Solution-Processed Silver Nanowire Electrodes. Adv. Mater..

[16] Gaynor W, Lee J-Y, Peumans P (2010). Fully Solution-Processed Inverted Polymer Solar Cells with Laminated Nanowire Electrodes. ACS Nano.

[17] Zhang D (2017). Silver Nanowires for Reconfigurable Bloch Surface Waves. ACS Nano.

[18] Li J (2014). Healable Capacitive Touch Screen Sensors Based on Transparent Composite Electrodes Comprising Silver Nanowires and a Furan/Maleimide Diels–Alder Cycloaddition Polymer. ACS Nano.

[19] Karakawa M, Tokuno T, Nogi M, Aso Y, Suganuma K (2017). Silver Nanowire Networks as a Transparent Printable Electrode for Organic Photovoltaic Cells. Electrochemistry.

[20] Rycenga M (2011). Controlling the synthesis and assembly of silver nanostructures for plasmonic applications. Chem. Rev..

[21] Piazza L (2015). Simultaneous observation of the quantization and the interference pattern of a plasmonic near-field. Nat. Commun..

[22] Kawata S (2013). Plasmonics for Nanoimaging and Nanospectroscopy. Appl. Spectrosc..

[23] Dregely D (2014). Imaging and steering an optical wireless nanoantenna link. Nat. Commun..

[24] Davies M (2012). Synchronous Emission from Nanometric Silver Particles through Plasmonic Coupling on Silver Nanowires. ACS Nano.

[25] Santoro G (2014). Silver substrates for surface enhanced Raman scattering: Correlation between nanostructure and Raman scattering enhancement. Appl. Phys. Lett..

[26] Bergin SM (2012). The effect of nanowire length and diameter on the properties of transparent, conducting nanowire films. Nanoscale.

[27] Xu Y (2017). Silver Nanowires Modified with PEDOT: PSS and Graphene for Organic Light-Emitting Diodes Anode. Sci. Rep..

[28] Hu H, Pauly M, Felix O, Decher G (2017). Spray-assisted alignment of Layer-by-Layer assembled silver nanowires: a general approach for the preparation of highly anisotropic nano-composite films. Nanoscale.

[29] Yan Z (2012). Controlling the Position and Orientation of Single Silver Nanowires on a Surface Using Structured Optical Fields. ACS Nano.

[30] Wang J (2015). A highly sensitive and flexible pressure sensor with electrodes and elastomeric interlayer containing silver nanowires. Nanoscale.

[31] Lee H, Kim M, Kim I, Lee H (2016). Flexible and Stretchable Optoelectronic Devices using Silver Nanowires and Graphene. Adv. Mater..

[32] Bobinger, M. *et al*. Solution processing of silver nanowires for transparent heaters and flexible electronics. In *2017 13th Conference on Ph*.*D*. *Research in Microelectronics and Electronics (PRIME)* 9–12, 10.1109/PRIME.2017.7974094 (IEEE, 2017).

[33] Miller MS, O’Kane JC, Niec A, Carmichael RS, Carmichael TB (2013). Silver Nanowire/Optical Adhesive Coatings as Transparent Electrodes for Flexible Electronics. ACS Appl. Mater. Interfaces.

[34] Liu Z, Xu J, Chen D, Shen G (2015). Flexible electronics based on inorganic nanowires. Chem. Soc. Rev..

[35] Valentine AD (2017). Hybrid 3D Printing of Soft Electronics. Adv. Mater..

[36] Sun Y, Gates B, Mayers B, Xia Y (2002). Crystalline Silver Nanowires by Soft Solution Processing. Nano Lett..

[37] Korte KE, Skrabalak SE, Xia Y (2008). Rapid synthesis of silver nanowires through a CuCl- or CuCl 2 -mediated polyol process. J. Mater. Chem..

[38] Bailey WJ (1973). Synthesis of Monomers That Expand on Polymerization. J. Elastoplast..

[39] Ji L, Chang W, Cui M, Nie J (2013). Photopolymerization kinetics and volume shrinkage of 1,6-hexanediol diacrylate at different temperature. J. Photochem. Photobiol. A Chem..

[40] Hu W (2012). Intrinsically stretchable transparent electrodes based on silver-nanowire–crosslinked-polyacrylate composites. Nanotechnology.

[41] Zeng X-Y, Zhang Q-K, Yu R-M, Lu C-Z (2010). A New Transparent Conductor: Silver Nanowire Film Buried at the Surface of a Transparent Polymer. Adv. Mater..

[42] Yu Z, Li L, Zhang Q, Hu W, Pei Q (2011). Silver Nanowire-Polymer Composite Electrodes for Efficient Polymer Solar Cells. Adv. Mater..

[43] Xu W (2018). Silver Nanowire-Based Flexible Transparent Composite Film for Curvature Measurements. ACS Appl. Nano Mater..

[44] Sun Y, Mayers B, Herricks T, Xia Y (2003). Polyol Synthesis of Uniform Silver Nanowires: A Plausible Growth Mechanism and the Supporting Evidence. Nano Lett..

[45] Lofton C, Sigmund W (2005). Mechanisms Controlling Crystal Habits of Gold and Silver Colloids. Adv. Funct. Mater..

[46] Xia Y, Xiong Y, Lim B, Skrabalak SE (2009). Shape-Controlled Synthesis of Metal Nanocrystals: Simple Chemistry Meets Complex Physics?. Angew. Chemie Int. Ed..

[47] Lazzari R (2002). IsGISAXS: a program for grazing-incidence small-angle X-ray scattering analysis of supported islands. J. Appl. Crystallogr..

[48] van der Pauw LJ (1958). A method of measuring the resistivity and Hall coefficient on lamellae of arbitrary shape. Philips Technical Review.

[49] Hexemer A, Müller-Buschbaum P (2015). Advanced grazing-incidence techniques for modern soft-matter materials analysis. IUCrJ.

[50] Roth SV (2016). A deep look into the spray coating process in real-time—the crucial role of x-rays. J. Phys. Condens. Matter.

[51] Schwartzkopf M, Roth S (2016). Investigating Polymer–Metal Interfaces by Grazing Incidence Small-Angle X-Ray Scattering from Gradients to Real-Time Studies. Nanomaterials.

[52] Buffet A (2012). P03, the microfocus and nanofocus X-ray scattering (MiNaXS) beamline of the PETRA III storage ring: the microfocus endstation. J. Synchrotron Radiat..

[53] Benecke G (2014). A customizable software for fast reduction and analysis of large X-ray scattering data sets: applications of the new DPDAK package to small-angle X-ray scattering and grazing-incidence small-angle X-ray scattering. J. Appl. Crystallogr..

