# Supplementary material for: Functional Printing of Conductive Silver-Nanowire Photopolymer Composites
Source: Sci Rep. 2019 Apr 23;9:6465. doi: 10.1038/s41598-019-42841-3 (PMC6478917; doi:10.1038/s41598-019-42841-3)
Supplement: Supplementary file 1 — Supplementary Information [file 41598_2019_42841_MOESM1_ESM.pdf]

Supporting Information

Functional Printing of Conductive Silver-Nanowire Photopolymer Composites

Tomke E. Glier[1], Lewis Akinsinde[1], Malwin Paufler[1], Ferdinand Otto[1], Maryam Hashemi[1], Lukas Grote[1], Lukas Daams[1], Gerd Neuber[1], Benjamin Grimm-Lebsanft[1], Florian Biebl[1], Dieter Rukser[1], Milena Lippmann[2], Wiebke Ohm[2], Matthias Schwartzkopf[2], Calvin J. Brett[2,3,4], Toru Matsuyama[5], Stephan V. Roth[2,6]*, and Michael Rübhausen[1]**

[1]Institut für Nanostrukturforschung, Center for Free Electron Laser Science (CFEL), Universität Hamburg, Germany.

[2]DESY, Notkestrasse 85, 22607 Hamburg, Germany.

[3]KTH Royal Institute of Technology, Department of Mechanics, Teknikringen 8, 100 44 Stockholm, Sweden.

[4]Wallenberg Wood Science Center, Teknikringen 56-58, 100 44, Stockholm, Sweden.

[5]Max-Planck Institute for the Structure and Dynamics of Matter, Luruper Chaussee 149, 22761 Hamburg, Germany.

[6]KTH Royal Institute of Technology, Department of Fiber and Polymertechnology, Teknikringen 56-58, 100 44 Stockholm, Sweden.

Corresponding Authors

*tglier@physnet.uni-hamburg.de; ruebhausen@physnet.uni-hamburg.de; stephan.roth@desy.de

S1. Sample Preparation

S1.1. Cleaning of the Substrates

Microscope slides from VWR company (transparent float glass, 76 x 26 mm, thickness = 1 mm) were used as substrates for transmission and conductivity measurements. The samples for GISAXS measurements were prepared on silicon wafers from Si-Mat (<100>, p-type, doped with boron). The glass slides and silicon wafers were cleaned with acetone (Carl Roth, ≥ 99.5 %) (ultrasonic bath, 5 min). Subsequently they were rinsed with isopropanol (Emsure, ≥ 99.8 %) and ultra-pure water (18 MΩ/cm) and dried with nitrogen. For the preparation of GISAXS samples without polymer coating the silicon substrates (13 x 13 mm^2) were cleaned using Piranha cleaning (95 % H_2SO_4, 30 % H_2O_2, ratio: 7:1)[1] instead of the organic cleaning with acetone and isopropanol, to achieve a better wetting of the silicon surface with the Ag-NW suspension.

S1.2. Deposition of Silver Nanowires

Different techniques for the deposition of silver nanowires (Ag-NW) on the substrate were explored. *Via* dip coating non-uniform Ag-NW layers without a complete percolation and with uncovered areas were produced (Fig. S1 (a)). Spin coating leads to Ag-NW layers with an inhomogeneous distribution and agglomeration in the middle of the sample. Droplets of the dispersion, which are spun to the edge of the sample, produce grooves in the Ag-NW layer (Fig. S1 (b)). By blading with a glass rod, the major part of the Ag-NW were aligned in blading direction and no percolation network could be achieved (Fig. S1 (c)). The best results were achieved by drop casting. After deposition, the drop was fully dried at room temperature for about 3 min. The solvent evaporates from the outside to the inside and a ring with a high Ag-NW concentration

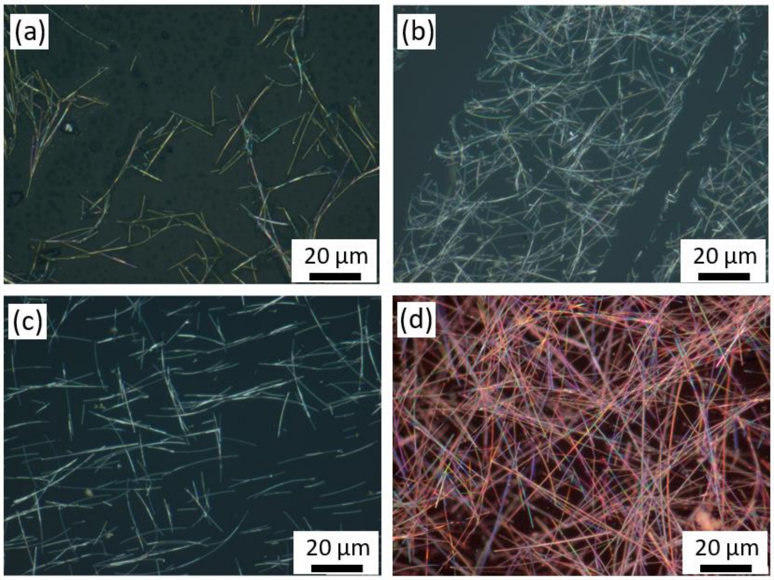

Fig. S1: Light microscopy images (Olympus light microscope BX 51) of (a) Ag-NW applied *via* dip coating, (b) Ag-NW layer produced by spin coating, (c) Ag-NW applied with a glass rod and (d) Ag-NW percolation network fabricated *via* drop casting.

at the edge was observed. In the middle of the samples, uniform Ag-NW percolation networks could be produced (Fig. S1 (d)). By using a frame of 3 cm^2 as a template during the drop casting process, an Ag-NW layer with well defined area could be produced.

S1.3. Argon Plasma Treatment

For the plasma treatment with Argon a Harrick Plasma Cleaner PDC-002 (230 V) was used at 22 °C. Before starting the Argon stream, the sample chamber was evacuated to a vacuum in the range of 1.7×10^{-4} – 3.2×10^{-4} mbar. During the plasma treatment, the pressure with flow of Argon in the chamber was between 1.0×10^{-2} and 1.2×10^{-2} mbar.
By using Argon plasma treatment, the polyvinylpyrrolidone (PVP) shell, which stabilizes the nanowires during the synthesis, was removed resulting in a decrease in sheet resistance (see inset of Fig. S2 (a)). The PVP shell can be clearly seen in the SEM image shown in Fig. S2 (a). At the interconnects of the Ag-NWs, partial welding occur due to the Argon plasma treatment.[2,3] Fig. S2 (b) and (c) shows SEM images of plasma treated Ag-NWs. Additionally, defects like holes are visible in the Ag-NW surface.[4,5]

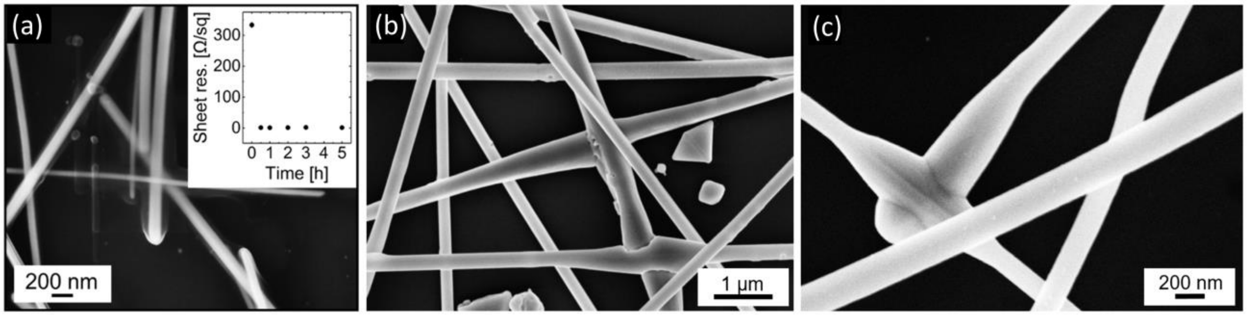

Fig. S2: (a) Ag-NWs with polyvinylpyrrolidone (PVP) shell (Ag-NW density around 50 µg/cm^2). Inset: Sheet resistance at a concentration of 58 µg/cm^2 as a function of plasma treatment duration; a drastic drop is observed after even the shortest treatment of 30 min. (b) and (c) Nanowire junctions after plasma treatment (concentration around 50 µg/cm^2).

S1.4. UV-curable Resin

For the optimization of the resin the concentrations of the different components (initiator, monomer and cross-linker) were varied. The aim was the minimization of the layer thickness and surface roughness. A cross-linker was added to increase the viscosity and reduce the sedimentation. Beside the fabrication of layered structures (sandwich structure: polymer-Ag-NW-polymer), Ag-NWs were mixed with the polymer and the filler material could be kept in

suspension for several hours. Fig. S3 shows a light microscopy image of the produced Ag-NW-polymer composite.

For the resin the following chemicals were used: Dipentaerythritol penta-/hexa-acrylate (DPPHA, cross-linker, Sigma-Aldrich, 5 mL), Phenylbis(2,4,6-trimethylbenzoyl) phosphine oxide (BAPO, initiator, Sigma-Aldrich, 0.33 g) and 1,6-Hexanediol diacrylate (HDDA, Sigma-Aldrich, 3 mL). First, HDDA was mixed with BAPO and stirred at 40 °C for 15 min. Subsequently, the cross-linker was added and the mixture was stirred until a homogeneous resin was achieved. The used chemical components are listed in Tab. S1.

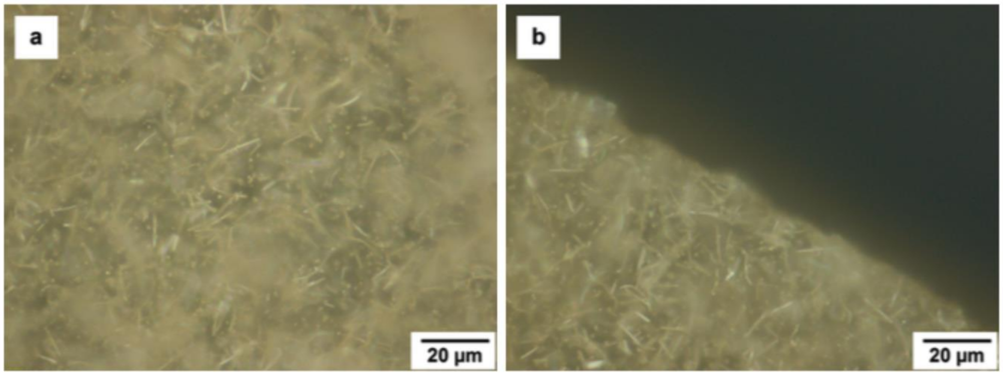

Fig. S3: Top view (a) and cross section (b) of a silver nanowire composite (4 %wt silver). The composite was cured in the illumination setup (Fig. S4).

Tab. S1: Components of the used resin and their molecular formula und molecular weight.

	**Monomer**	**Cross-linker**	**Initiator**
**Structural chemical formula**	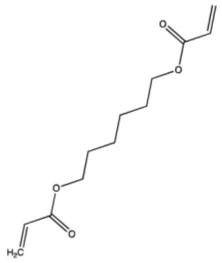	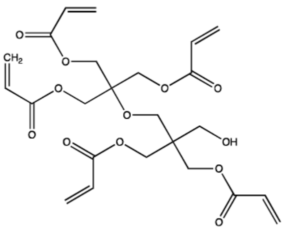	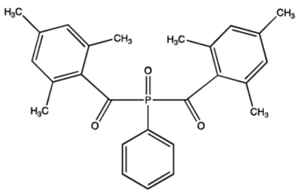
**Substance**	1,6-Hexanediol diacrylate<br><br>HDDA	Dipentaerythritol penta-/hexa-acrylate<br><br>DPPHA	Phenylbis(2,4,6-trimethylbenzoyl) phosphine oxide<br><br>BAPO
**Molecular Formula**	$C_{12}H_{18}O_4$	$C_{25}H_{32}O_{12}$	$C_{26}H_{27}O_3P$
**Molecular Weight**	226.27 g/mol	524.51 g/mol	4.18.46 g/mol

S1.5. Curing Setup

The resin was cured in a curing setup - a schematic representation is shown in Fig. S4. A Coherent BioRay laser with a wavelength of 405 nm and a power of 52 mW was used. The used DC voltage was 5 V. The power was measured with a Field master power meter. Directly behind the collimated laser the power was equal to 46.8 mW and behind the diffuser a power of 3.4 mW was achieved. A shutter controller (Thorlabs SC-10), shutter (Thorlabs SH-05) and beam expander (Thorlabs BE05M-A) were used. Samples with silicon substrate were exposed from above.

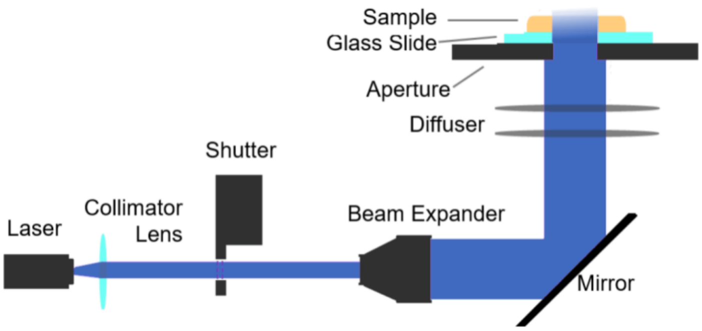

Fig. S4: Schematic illustration of the curing setup.

S1.6. Sample Overview

Tab. S2 shows a summary of the produced Ag-NW networks and composite materials and their key properties like sheet resistance, transmission, film thickness and roughness. Film thickness and roughness were determined by profilometry (S2.2).

S2. Characterization Methods

S2.1. Scanning Electron Microscopy (SEM) and Energy Dispersive X-ray Spectroscopy (EDX)

The study of the surface morphology and shape of the samples were carried out on a commercial field effect scanning electron microscopy (FESEM) equipment from Zeiss company. For a bright resolution image, an acceleration voltage of 15 kV as well as a ca. 6 mm focal distance of the lenses with resulting magnification of 80000 x were used. Surface sensitive measurements were carried out with an acceleration voltage of 2-5 kV. The mean length and mean diameter of the Ag-NW were determined and histograms were created *via* evaluation of the SEM images. In Fig. S5, histograms of the diameters and of the Ag-NW length are shown as an example. Fig. S6 shows SEM images of silver nanowires. The pentagonal structure and fivefold twinned tip of the Ag-NW were verified.

Tab. S2: Sample overview

Sample	Ag-NW density [µg/cm²]	Sheet Resistance [Ω/sq]		Transmission (λ= 600 nm) [%]		Film Thick-ness [µm]	Rough-ness [nm]
		Ag-NW	Composite	Ag-NW	Composite	Ag-NW	Composite
A-1	26	576 ± 56	101.0 ± 0.6	92.0 ± 0.5	94.0 ± 0.5	107 ± 11	110 ± 3
A-2	26	143 ± 26	60.0 ± 0.1	92.0 ± 0.5	94.0 ± 0.5	107 ± 11	110 ± 3
A-3	26	711 ± 85	374 ± 8	92.0 ± 0.5	94.0 ± 0.5	107 ± 11	110 ± 3
A-4	39	75.00 ± 0.04	28.0 ± 0.1	90.0 ± 0.5	92.0 ± 0.5	107 ± 11	110 ± 3
A-5	39	222 ± 8	48.0 ± 0.1	90.0 ± 0.5	92.0 ± 0.5	107 ± 11	110 ± 3
A-6	39	202 ± 2	78.0 ± 0.1	90.0 ± 0.5	92.0 ± 0.5	107 ± 11	110 ± 3
A-7	65	20.00 ± 0.02	13.00 ± 0.01	88.0 ± 0.5	90.0 ± 0.5	107 ± 11	110 ± 3
A-8	65	25.00 ± 0.01	17.00 ± 0.04	88.0 ± 0.5	90.0 ± 0.5	107 ± 11	110 ± 3
A-9	65	25.00 ± 0.01	19.00 ± 0.03	88.0 ± 0.5	90.0 ± 0.5	107 ± 11	110 ± 3

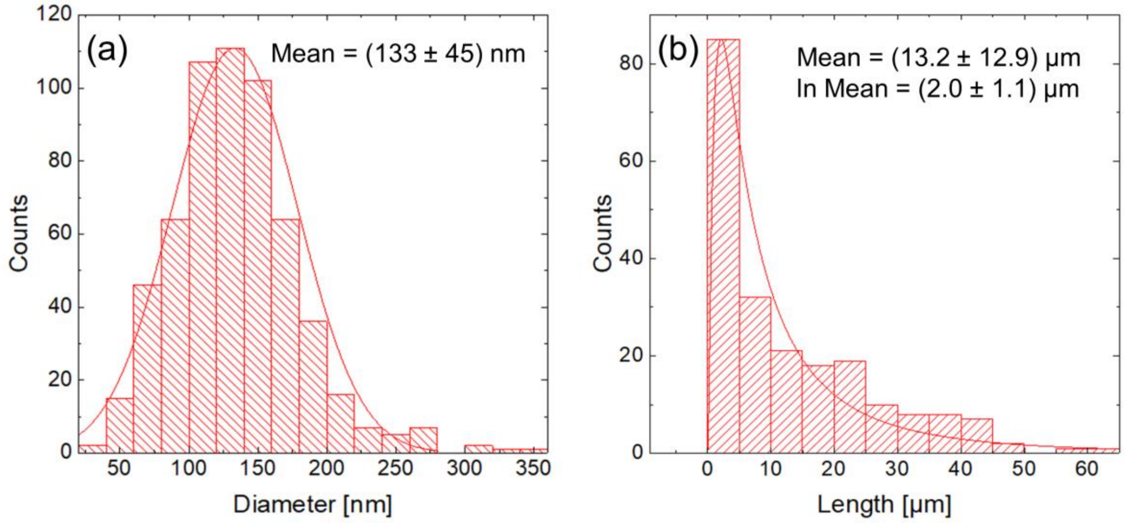

Fig. S5: Histograms of the diameter (a) and length (b) of the synthesized silver nanowires used for GISAXS. The diameter distribution (a) was fitted with a Gaussian distribution and the distribution of the Ag-NW length (b) follows a lognormal distribution.

The chemical composition of the silver nanowire samples was investigated *via* energy dispersive X-ray spectroscopy (EDX, Zeiss). The spectrum of a plasma untreated Ag-NW percolation network shows a silicon peak (substrate) and silver peaks (Ag-NW). Additionally, carbon and oxygen were detected because of the presence of polyvinylpyrrolidone (PVP, stabilizing ligand) (Fig. S7 (a)). After argon plasma treatment (5 h), the PVP shell was completely removed and no carbon and oxygen were detected by EDX (Fig. S7 (b)).

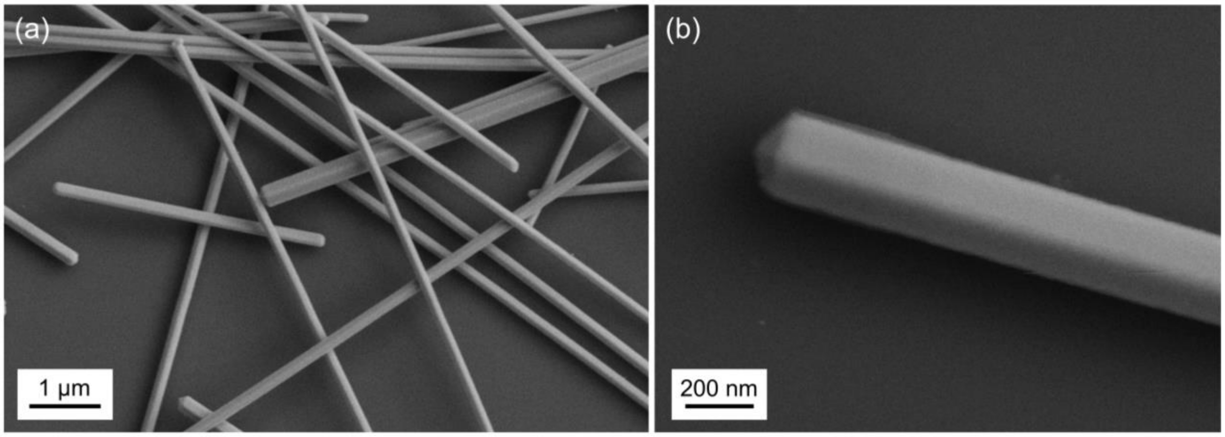

Fig. S6: (a) SEM images of silver nanowires (acceleration voltage = 5 kV, working distance = 8.4 mm, SE2 detector). (b) The pentagonal structure and the fivefold twinned tip of the silver nanowires are clearly visible.

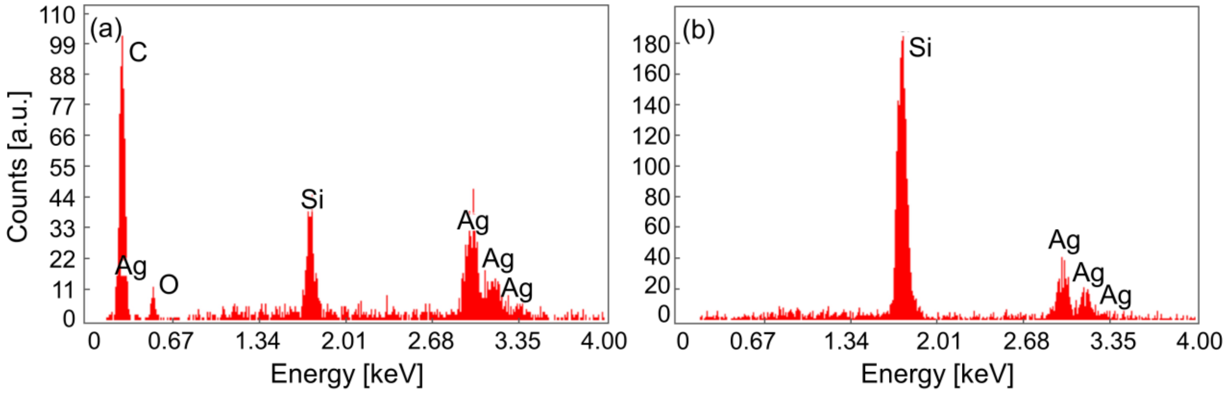

Fig. S7: (a) EDX spectrum of a plasma untreated Ag-NW network. Silicon (substrate), silver (Ag-NW) and carbon and oxygen (PVP) were detected. (b) After 5 hours plasma treatment with argon, no PVP as indicated by the absence of carbon and oxygen was identified.

S2.2. Profilometry and Atomic Force Microscopy (AFM)

Profilometry measurements were performed with a Dektak XT equipment from Bruker company to determine layer thickness and surface roughness. Furthermore, the surface topography of the samples was investigated with an NT-MDT Solver NEXT tabletop scanning probe microscope in

semi contact (tapping) mode and an Anfatec Level AFM in tapping mode. Fig. S8 (a) shows an AFM image of a silver nanowire percolation network (c = 58 µg/cm^2) on a silicon substrate. The Ag-NW layer thickness is ~600 - 700 nm. Exemplary AFM images for a pure polymer layer, an Ag-NW polymer composite and a sandwich sample are shown in Fig. S8 (b)-(d). The mean roughness (R_a) and root-mean-squared roughness (R_{ms}) could be determined. The resulting roughnesses are similar to the mean roughnesses determined *via* profilometry measurements of the same samples (compare Fig. 1 (c) of the main text). *Via* AFM, a higher resolution was achieved, but a smaller sample area could be measured.

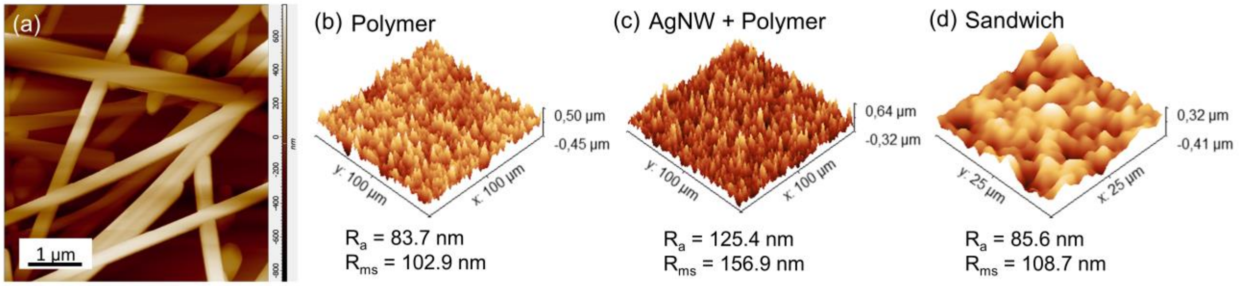

Fig. S8: (a) AFM image of an Ag-NW percolation network (c = 58 µg/cm^2). (b)-(d) AFM images of polymer samples (polymer reference, polymer composite, and sandwich). The determined mean roughnesses (R_a) and root-mean-squared roughnesses (R_{ms}) are indicated.

S2.3. Conductivity Measurements (Van-der-Pauw)

For resistivity measurements in Van der Pauw geometry, DPP 105-M/V-Al-S positioners (CascadeMicrotech, USA), a DC voltage/current source GS200 (Yokogawa, Japan) and the 34401A 6 ½ Digit Multimeter (Keysight, USA) were used. Four points of silver lacquer were applied on the edge of the sample in order to achieve a good contact to the Ag-NW network. An input current was applied at the points A and B (Fig. S9) and was varied between -0.6 mA and 0.6 mA. The voltage drop at the points on the opposite side (C and D) was measured. The measurement was repeated with an input current at A and D and the voltage was measured between B and C. The sheet resistance was calculated according to the Van der Pauw approximation[6]. A photograph of the setup is shown in Fig. S9 (b).

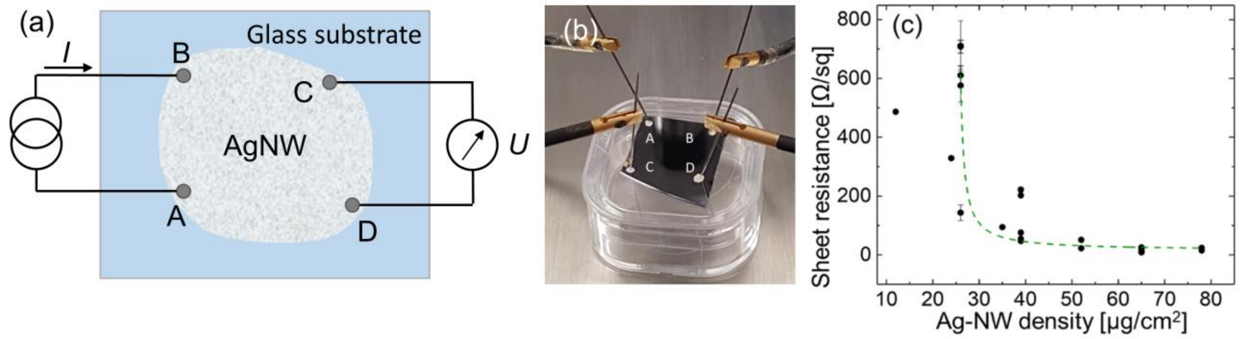

Fig. S9: (a) Schematic illustration of the Van der Pauw geometry. Ag-NW were coated on a glass substrate and the Ag-NW network was contacted with conductive silver lacquer (A, B, C, and D). (b) Photograph of the setup for resistivity measurements in Van der Pauw geometry. Instead of an Ag-NW sample a silicon wafer is shown. The contacts (silver lacquer) are labelled with A, B, C and D). (c) Sheet resistance of washed Ag-NWs (see details in main text) on substrate as function of Ag-NW surface density. Please note the critical behavior around (25 ± 10) µg/cm^2. The green line is a guide to the eye. Each point represents one deposition/measurement.

As seen in Fig. S9 (c) showing our results of washed Ag-NW samples, we find a critical nanowire concentration of (25 ± 10) µg/cm^2 above which we reproducibly find conducting nanowire networks. In this range, the sheet resistance drops dramatically reaching values of down to 10 Ω/sq. At the critical nanowire concentration, we also observe some fluctuations in sheet resistance, which relate to the fact that at low concentration the probability of good electrical connection decreases and sensitivity towards the exact results of the washing procedure increases. However, the Ag-NW network showing a sheet resistance of around 500 Ω/sq at a concentration of only 10 µg/cm^2 indicates that with further optimization better conductivities at even lower Ag-NW concentration could be feasible.

S2.4. Ellipsometry

In addition to the characterization methods mentioned in the main text, ellipsometry was used to investigate the homogeneity and anisotropy of silver nanowire layers. A spectroscopic ellipsometer (SE-850) from Sentech was used. The following three samples were investigated: W1 (15 µL Ag-NW suspension), W2 (2 x 15 µL Ag-NW suspension) and W3 (3 x 15 µL Ag-NW suspension). For the samples W2 and W3 a second (and a third) Ag-NW layer was applied after the previous layer was dried. The homogeneity test was carried out at an incidence angle of 70° and different beam diameters. The phase difference Δ was determined. In Fig. S10 (a) the difference spectra are shown. Sample W1 (one layer Ag-NW) is homogeneous, sample W2 (2 layers) shows a slight inhomogeneity and W3 (3 layers) a significant inhomogeneity. To

investigate the anisotropy, the samples were rotated 45° for the second measurement. The incidence angle was 70° and the beam diameter was kept constant. The difference spectra are shown in Fig. S10 (b). Sample W1 is nearly isotropic. With increasing layer number, the anisotropy is increasing.

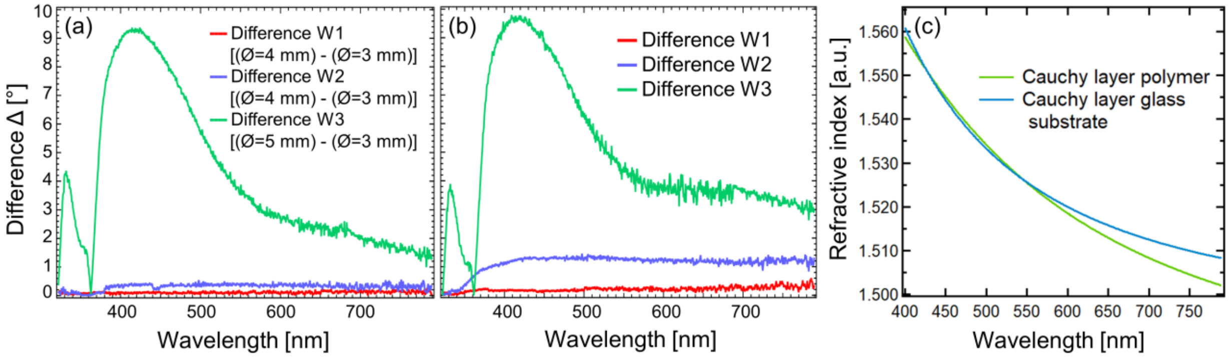

Fig. S10: (a) Difference of measured phase difference Δ for different beam diameters for the samples W1 (1 x 15 µL Ag-NW suspension), W2 (2 x 15 µL Ag-NW suspension) and W3 (3 x 15 µL Ag-NW suspension). (b) Difference of measured phase difference Δ for different sample orientations. The samples (W1, W2 and W3) were rotated 45° for the second measurement. (c) Refractive index of a polymer layer on a glass substrate (green) and of a glass substrate (blue) determined by fitting the data with a Cauchy model (fitting parameters glass: $N_0 = 1.494$, $N_1 = 82.8$, $N_2 = 38.7$, $K_0 = 0.000$, $K_1 = 8.214$, $K_2 = 16.417$; polymer: $N_0 = 1.477$, $N_1 = 164.5$, $N_2 = -53.8$, $K_0 = -0.001$, $K_1 = 5.023$, $K_2 = 6.409$).

Additionally, the optical properties of a polymer layer (S1.4.) on a glass substrate were investigated by ellipsometry. A thin and homogeneous (homogeneity test with different beam diameters) polymer layer was produced by rod coating. The data were fitted with a Cauchy model and the determined layer thickness is equal to 881 nm. Fig. S10 (c) shows the refractive index determined with the Cauchy model[7] of the polymer sample in comparison to a bare glass substrate.

S2.5. Grazing Incidence Small Angle X-ray Scattering (GISAXS)

GISAXS measurements were carried out at the micro- and nanofocus X-ray scattering beamline MiNaXS/P03 at PETRA III (DESY)[8]. We used a sample-detector-distance (SDD) of 4990 ± 1 mm (polymer sample: 3600 ± 1 mm) and a wavelength of 0.9724 Å. The schematic illustration of the GISAXS setup is shown in Fig. S11 (a). The direct beam was located at x = 545 pixel and y = 15 pixel (polymer sample: x = 532 pixel, y = 72 pixel). The incidence angles are listed in Tab. S3. After height, tilt and angle adjustment, the samples were scanned through the beam with a step size of 50 µm and an acquisition time of 1 s in order to avoid radiation damage (x-scan). An example for a stack plot of the GISAXS cuts at y = 365 pixel (corresponding to $q_z = 0.78\ nm^{-1}$, width = 10

pixel) is shown in Fig. S11 (c). In order to increase in statistics, all GISAXS images of one scan were summed up. The summed-up images are shown in the main text of this manuscript. For analysis of the data, we used the software DPDAK[9] (v1.3.0). The horizontal cuts were carried out at y = 300 pixel (q_{z1} = 0.63 nm^{-1}), y = 365 pixel (q_{z2} = 0.78 nm^{-1}) and y = 445 pixel (q_{z3} = 0.96 nm^{-1}) with a height of 10 pixel, respectively. By following the q_y-position of the peaks caused by the flares (arrows in Fig. 2(g)-(i)), an angle of the flares of around 36° was found. The positions of the horizontal cuts are marked in Fig. S11 (b).

Tab. S3: Incidence angles of the measured samples.

Fig. 2	Sample	Incidence angle
Fig. 2 (a)	Wires	0.527
Fig. 2 (b)	Composite	0.462
Fig. 2 (c)	Polymer	0.527
Fig. S12	Plasma treated wires (30 min)	0.539

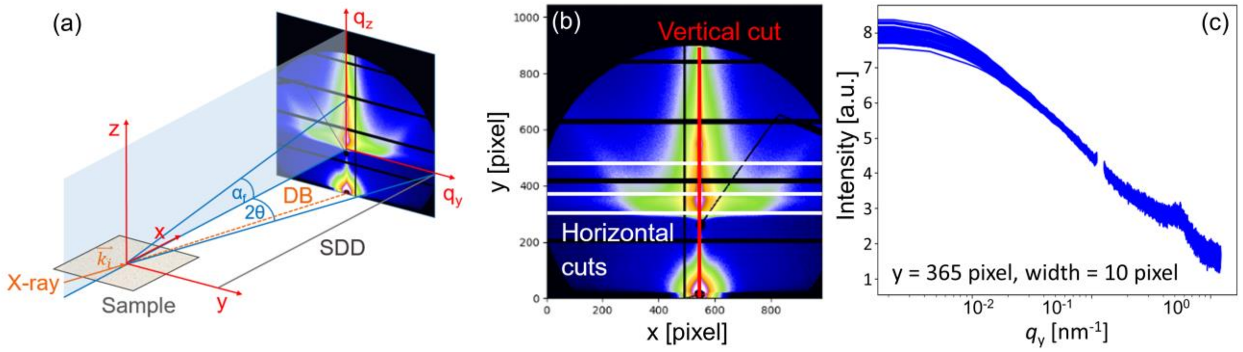

Fig. S11: (a) Schematic illustration of the GISAXS setup. The incoming X-ray beam ($\overrightarrow{k_i}$) impinges under an angle < 1° onto the sample surface. The scattered photons are collected with a 2D detector at a sample-to-detector distance SDD. x, y, z denotes a real space coordinate system and q_y and q_z a reciprocal space coordinate system. DB denotes the direct beam. (b) Summed GISAXS data of a silver nanowire network without plasma treatment. 51 images of a horizontal scan lateral to the beam direction on the sample were summed-up. The white lines represent the horizontal cuts at y = 300 pixel, y = 365 pixel and y = 445 pixel (height =10 pixel) and the red line represents the position of the vertical cut at x = 535 pixel (width = 20 pixel). (c) Stacked horizontal cuts of 51 images taken during the horizontal scan of an Ag-NW network without plasma treatment.

The influence of Argon plasma treatment on the nanowire geometry was studied using GISAXS. In Fig. S12, the flares have been strongly reduced due to the Argon plasma treatment of the Ag-NWs in comparison to Fig. 2(a) in the manuscript. Argon plasma treatment was used to remove the PVP layer and to improve the conductivity of the Ag-NW network (inset SI S1.3). However, during the plasma treatment, the surface of the Ag-NWs becomes rougher due to defects[4,5] and the shape more round due to the removal of edges.[10] Furthermore, at the interconnects of the Ag-NWs, partial welding and sputtering occurs[2,3] (see SI Fig. S2), which also gives rise to additional scattering from spherical structures as observed by the increased diffuse scattering in Fig. S12. Furthermore, plasma treatments are rather incompatible with a 3D-printing approach. For this reason, we concentrate on the removal of PVP through the controlled washing of the nanowires.

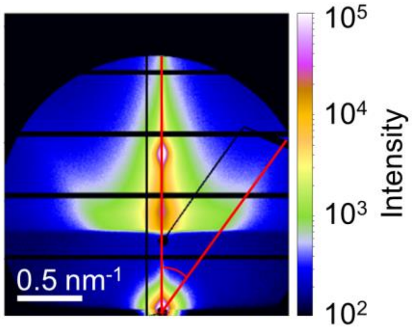

Fig. S12: Two-dimensional (2D) GISAXS pattern from a Ag-NW network (58 µg/cm^2, washed), which was treated with Argon plasma for 30 min.

S2.6. GISAXS Simulation

We used the software IsGISAXS[11] V1.6 for simulating the GISAXS pattern. The actual sample morphology in terms of thin film is a 3D percolated network of Ag-NW with a film thickness of ~600 nm. The mean diameter and mean length of an individual Ag-NW is 130 nm and 10 µm (see Fig. S5). In terms of simulation, we need to identify the key scattering features, which are in our case the Yoneda region and the flares at an angle of 36° with respect to the q_z-axis. As such flares usually indicate faceting, we opted for an anisotropic pyramid with an aspect ratio of 10:1 at a radius of R_0 = 60 nm in order to simplify calculations. The facet angle with respect to the sample surface was 36°. The object was oriented with the long axis along the beam (zeta = 0°). The radius distribution was Gaussian with a width of σ_R/R_0 = 0.7 between 3 nm and 90 nm. The height and width of the object were calculated using a Gaussian distribution: H/R_0 = 0.55, width = 0.7, range of H/R_0 = 0.55-1.5; W/R_0 = 10, width = 0.7, range of W/R_0 = 1-200. Thus, the mean values were H = 0.55 R_0 and W = 10 R_0. The structure factor was of one-dimensional paracrystalline type with a peak position of 390 nm and a Gaussian width of 190 nm. The value of D reflects the fact that a 3D network is present, where the Ag-NWs touch each other at interconnects (see Fig. S6), while at the same time the porosity is around 11%. Reflection and refraction effects were included by using the distorted-wave Born approximation (DWBA) (comparable to previous work, see [12]). This choice of parameters allowed for reproducing the key scattering features in a reasonable way.

The logarithmic intensity/color scale in Fig. 2 is chosen in such a way that the dynamic range (10^3) corresponds to that of the data (10^3).

S3. Literature

1. Müller-Buschbaum, P. Influence of surface cleaning on dewetting of thin polystyrene films. *Eur. Phys. J. E* **12,** 443–448 (2003).
2. Liu, L. *et al.* Nanowelding and patterning of silver nanowires via mask-free atmospheric cold plasma-jet scanning. *Nanotechnology* **28,** 225301 (2017).
3. Wang, R. *et al.* Plasma-induced nanowelding of a copper nanowire network and its application in transparent electrodes and stretchable conductors. *Nano Res.* **9,** 2138–2148 (2016).
4. Ra, H. W. *et al.* Ion bombardment effects on ZnO nanowires during plasma treatment. *Appl. Phys. Lett.* **93,** 1–4 (2008).
5. Li, J. *et al.* A flexible plasma-treated silver-nanowire electrode for organic light-emitting devices. *Sci. Rep.* **7,** 1–9 (2017).
6. van der Pauw, L. J. A method of measuring the resistivity and Hall coefficient on lamellae of arbitrary shape. *Philips Technical Review* **20,** 220–224 (1958).
7. Poelman, D. & Smet, P. F. Methods for the determination of the optical constants of thin films from single transmission measurements: a critical review. *J. Phys. D Appl. Phys* **36,** 1850–1857 (2003).
8. Buffet, A. *et al.* P03, the microfocus and nanofocus X-ray scattering (MiNaXS) beamline of the PETRA III storage ring: The microfocus endstation. *J. Synchrotron Radiat.* **19,** 647–653 (2012).
9. Benecke, G. *et al.* A customizable software for fast reduction and analysis of large X-ray scattering data sets: Applications of the new DPDAK package to small-angle X-ray scattering and grazing-incidence small-angle X-ray scattering. *J. Appl. Crystallogr.* **47,** 1797–1803 (2014).
10. Winkler, K., Wojciechowski, T., Liszewska, M., Górecka, E. & Fiałkowski, M. Morphological changes of gold nanoparticles due to adsorption onto silicon substrate and oxygen plasma treatment. *RSC Adv.* **4,** 12729–12736 (2014).
11. Lazzari, R. IsGISAXS: A program for grazing-incidence small-angle X-ray scattering analysis of supported islands. *J. Appl. Crystallogr.* **35,** 406–421 (2002).
12. Schwartzkopf, M. *et al.* Real-Time Monitoring of Morphology and Optical Properties during

Sputter Deposition for Tailoring Metal–Polymer Interfaces. *ACS Appl. Mater. Interfaces* **7,** 13547–13556 (2015).
